# Double Burden of Malnutrition: Evidence from a Selected Nigerian Population

**DOI:** 10.1155/2020/5674279

**Published:** 2020-09-01

**Authors:** Emmanuel O. Alamu, Toluwalope E. Eyinla, Rasaki A. Sanusi, Busie Maziya-Dixon

**Affiliations:** ^1^Food and Nutrition Sciences Laboratory, International Institute of Tropical Agriculture (IITA), Southern Africa Hub, P.O. Box 310142, Chelston, Lusaka, Zambia; ^2^Food and Nutrition Sciences Laboratory, International Institute of Tropical Agriculture (IITA), PMB 5320, Oyo Road, Ibadan, Oyo State, Nigeria; ^3^Department of Human Nutrition, College of Medicine, University of Ibadan, Ibadan, Oyo State, Nigeria

## Abstract

Indices reflecting the double burden of malnutrition in sub-Saharan Africa are increasing. Evidence to support this claim in households of Africa's most populous country—Nigeria—is scant. This study, therefore, presents results from a study of mother-child pairs sampled from Akwa Ibom State in the southern region of Nigeria. Anthropometric measures for 660 mother-child pairs were collected according to standard procedures. Indices were expressed as the standard deviation of units from the median for the reference group. Chi-square analysis was used to test significant differences in proportion, and *p* < 0.05 was taken as significant. A total of 37.4% of the children were stunted out of which 19.8% were moderately stunted, and 17.6% were severely stunted. Prevalence of wasting was 13.1%, 6.2% were moderately wasted, and 6.9% were severely wasted. Mean maternal body mass index was (23.54 ± 4.60) kgm^2^. 9.0% were underweight mothers, 23.2% were overweight, and 9.3% were obese. The co-existence of undernutrition among children and overnutrition in women of child-bearing age is prevalent in this population. We recommend that more effort be placed on active nutrition surveillance to ascertain malnutrition prevalence and periodically reassess priority challenges.

## 1. Introduction

One of the most critical global disease burdens is malnutrition presenting as deficiencies and excess [1]. This burden accounts for at least 9 million deaths per year in children less than five years of age [2]. It is a significant public health problem, most notably in developing countries [3–5] where 90% of the world's undernourished children live. More troubling is the co-existence of both undernutrition and overnutrition in the same population, which is currently becoming a significant health problem globally [5]. This trend is more challenging in developing countries that are still tackling endemic undernutrition causes and effects [6, 7]. Poor nutrition during the early formative years has been implicated to result in significant morbidity and mortality and delayed mental and motor developments [8]. In the long term, it has been linked to impairments of intellectual performance, work capacity, reproductive outcomes, and overall health [9]. Unfortunately, a significant cause is poor maternal nutritional status, which can harm the offspring, thus leading to a vicious cycle of malnutrition from one generation to another [3]. Thus, the nutritional status of mothers is essential both for her health and that of her baby. The consequences of poor maternal nutritional status are reflected in high infant and maternal morbidity and mortality, as described by Black et al. [3], Elshibly and Schmalisch [10], and Ugwa [11]. Nutritional deficiencies could manifest as either protein-energy malnutrition or micronutrient malnutrition (hidden hunger). Hidden hunger is much more difficult and expensive to assess. However, anthropometric measurements are practical and realistic tools for rapid assessment of the nutritional status of populations in developing countries and have been adapted in assessing both women and children [11–13]. Evidence has been established proving that the effects of undernutrition and overnutrition occurring concurrently in an individual, household, or population can be damaging [14]. Its occurrence in sub-Saharan Africa has been reviewed [3]; however, the evidence is still lacking from Africa's most populous country—Nigeria. In 2001–2003, the Nigerian food consumption and nutrition survey revealed 42% stunting among children under the age of 5 years, underweight was 25%, and wasting was 9% [15].

Similarly, according to the 2008 Nigerian National Demographic and Health Survey, 41% of Nigerian children less than five years were stunted, 23% were underweight, and 14% were wasted [16] (NDHS 2008). The NDHS 2013 [17] survey reflected a mild drop in these indices (especially stunting) but still gave reports of nutritional status that signify public health challenges. Scant studies have probed this challenge in mother-child pairs in Nigeria. A recent study examined the underlying causal factors of malnutrition in obese mother/stunted child pairs [18]. A review of the literature reveals several studies around Nigeria have investigated the challenge of protein-energy malnutrition in children [18–22] and women [23–25]. A study [26] presented results of nutritional parameters in mother-child pairs in Akwa Ibom State, Nigeria, with the sole aim of probing vitamin A intake to justify the introduction of biofortified cassava (rich in carotenoids which is a vitamin A precursor) considering that the state has the highest consumption of Nigeria's chief carbohydrate crop—cassava. While this study expatiated on hidden hunger rates in the selected population, anthropometric information was not critically reported in this population. A closer look into these anthropometric indicators is justified by results of the Nigeria demographic health surveys in the past decade that have established that women in the southern regions were having highest rates of overnutrition which had increased from 26.7% in 2008 [16] to 32.05% in 2013 [17]. As have been found in similar populations [7, 18], these increases suggest that in the same household where the child may be undernourished, the mother (or primary caregiver) may be experiencing another form of malnutrition. This study was thus conducted to assess anthropometric indices among women of child-bearing age and children 6–59 months old in Akwa Ibom State, South-South region, Nigeria.

## 2. Methodology

### 2.1. Study Design

This was a descriptive cross-sectional study to assess anthropometric indices of women of child-bearing age and children aged between 6 and 59 months. It was conducted alongside the study on vitamin A status of women of child-bearing age in Akwa Ibom State, Nigeria.

### 2.2. Study Location

Akwa Ibom State, which is located in the South-South geopolitical zone and the humid forest agroecological zone, was selected as the project state. Earlier surveys had established that the state has the highest consumption of cassava and a high prevalence of vitamin A deficiency.

### 2.3. Sample Size Estimation

The estimation of sample size for this study was based on the prevalent data on vitamin A deficiency (VAD) and iron and zinc deficiencies obtained for the state from the National Nutrition survey [15]. The prevalence of wasting, stunting, and underweight obtained from the National Demographic and Health Survey [16] was included in the estimation of sample size. The sample size was calculated based on the following criteria and assumptions:

- The confidence level of 93.5 (precision of 6.5%)
- Power of 80%
- Estimated malnutrition among children of 30%
- A design effect of 2.5
- The individual response rate of 85%
- Seven individuals per household on average
- Sixteen percent of the population being children of 6–59 months of age
- A sample size of 660 households was estimated

Thus, a total of 660 households with women of child-bearing age and children of 6–59 months of age were selected for the study.

### 2.4. Sampling Procedures

The sample was selected using a multistage selection scheme consisting of three levels: selection of local government areas (LGAs), enumeration areas (EAs), and households. Akwa Ibom State has 31 LGAs, made up of 16 rural, 5 urban, and 10 periurban areas. In Nigeria, the current official designation of rural, urban, and periurban is based mainly on population. According to the National Population Commission of Nigeria, a community with less than 5,000 people is regarded as rural, between 5,000 and 20,000 people is regarded as periurban, and above 20,000 is regarded as urban. Since malnutrition is prevalent in both urban and rural centres and dietary habits cut across all sectors of urbanization, ten (10) LGAs were selected using the probability proportionate to size, such that the likelihood of an LGA being selected was proportionate to its size. This resulted in the selection of 5 rural, 1 urban, and 4 periurban areas. A random selection of three EAs within each LGA was made. Therefore, a total of 30 EAs were sampled. At least 22 households were sampled randomly at the community level from each EA totalling 660 households.

### 2.5. Anthropometry

Anthropometric indicators for women and children were collected in the study to provide outcome measures for nutritional status. Weight and height for both mothers and children were collected according to standard procedures, which included tared weighing procedure and length (recumbent) measurement for children under 24 months, while height measurement (standing) was carried out for children above 2 years and their mothers [16].

### 2.6. Data Processing and Statistical Analysis

Data entry was done using MS Access and MS Excel. Data verification, screening, and editing were carried out to ensure that the entry errors were corrected. Double entered data were compared using the compare procedure of the Statistical Analysis System (SAS) to identify erroneously recorded data which usually cannot be easily verified or corrected. Weight and height values were used to calculate and classify body mass index (BMI) for mothers based on the World Health Organization classifications. Mothers within the teenage category were classified using WHO-Anthro Plus software, 2006, using the BMI-for-age classification [27]. Height-for-age, weight-for-age, and weight-for-height were determined using WHO-Anthro Plus software, 2006. The results obtained were compared with reference values from the population of well-nourished children. Indices were expressed as the standard deviation of units from the median for the reference group.

Outlier values such as implausible values for anthropometric indices were excluded from the dataset. This accounts for variation in reported frequencies. Frequency of each variable was conducted to ensure that values are within the acceptable range. Essential basic descriptive statistics and plots on distribution were conducted using SAS version 9.2, Cary, NC, USA. The chi-square test tested the significance of differences in proportion, and *p* < 0.05 was taken as significant.

### 2.7. Ethics Approval and Informed Consent

Ethical clearance was obtained through the Nutrition Division in the National Health Research Ethics committee based in the Federal Ministry of Health, Abuja. Ethical approval was also obtained from Akwa Ibom State Research Ethics committee in the Ministry of Health. Written informed consent was obtained from the women who participated in the study after the study objectives had been explained.

## 3. Results

As presented in Table 1, a total of 547 children had data on age and mean ± standard deviation. The mean age among children was 30.84 ± 13.02 months. The number of boys was 273 with a mean age of 31.61 ± 13.38 months while the girls numbered 274, with a mean age of 30.08 ± 12.62 months. There was no significant difference in the mean age between boys and girls (*p*=0.17). Mean weight among children was 12.70 ± 3.78 kg, that of boys was 13.03 ± 3.76 kg, and girls had a mean weight of 12.36 ± 3.76 kg. There was a significant difference in mean weight between boys and girls (*p*=0.041). Five hundred and forty-three children had data on height, and the mean value was 87.28 ± 13.39 cm. Mean height for boys was 88.29 ± 14.57 cm and that of girls was 86.28 ± 12.04 cm. As presented in Table 2, a total of five hundred and ninety-six children were assessed for height-for-age; of these, 295 (49.5%) were boys, and 301 (50.5%) were girls. Three hundred and seventy-three (62.9%) children had normal height-for-age; of these, 183 (30.7%) were boys, and 190 (31.9%) were girls. The total prevalence of stunting among children was 223 (37.4%); of these, 112 (18.6%) were boys, and 111 (18.7%) were girls. Prevalence of mild/moderate stunting was 118 (19.8%), and that of severe stunting was 105 (17.6%). There was no significant difference in stunting between boys and girls. A total of 593 children were assessed for weight-for-age; of these, 294 (49.6%) were boys, and 299 (50.4%) were girls. The total prevalence of underweight among children was 108 (18.2%), comprising 67 (11.3%) moderate underweight and 41 (6.9%) severe underweight. Of the total number of underweight children, 53 (9.0%) were boys, and 55 (9.2%) were girls. There were no significant differences in underweight between boys and girls (*p* > 0.05). A total of five hundred and fifty-one children were assessed for weight-for-height. Results showed that a total of 72 (13.1%) of the children were wasted. Of these, 34 (6.2%) were moderately wasted, and 38 (6.9%) were severely wasted. Again, there was no significant difference between boys and girls in wasting. As presented in Table 3, the mean age among mothers was 27.28 ± 6.85. Mean weight and height were 58.43 ± 12.05 kg and 157.52 ± 6.56 cm, respectively. Anthropometric data from 620 women were used to calculate maternal BMI. Highest body mass index (BMI) was 45.45 kg/m^2^; the lowest BMI was 14.55 kg/m^2^, and the mean BMI was 23.54 ± 4.60 kg/m^2^. A total of 58.71% of the women had BMI values in the normal range. About 55 (8.87%) of the respondents were underweight, and 142 (23.39%) were overweight. Also, 57 (9.03%) were obese with a BMI higher than 30 kg/m^2^.  Table 4 presents the crosstabulation of mothers' BMI status in comparison with children's anthropometric indices. The mother-child comparison showed that 105 (16.94%) moderately stunted and 94 (15.16%) severely stunted children had 45 (8.25%) and 18 (2.90%) overweight or obese mothers, respectively. Among the 44 (7.13) children who were severely underweight, only 11 (1.78) and 4 (0.65) had overweight and obese mothers, respectively. For the weight-for-height index, 30 (4.89%) experiencing mild wasting and 37 (6.04%) of the children who were severely wasted had 15 (2.45%) overweight and 7 (1.14%) obese mothers. There was no significant (*p* > 0.055) relationship between mother's BMI and any of the children's anthropometric indices.

## 4. Discussion

This study thus presents information on the occurrence of double burden malnutrition after assessing anthropometric assessments in mother-child pairs living in Akwa Ibom State. The result of this study indicated a high prevalence of stunting and wasting among the children in Akwa Ibom State. The underweight levels were, however, of medium severity. The stunting levels in this study were lower than a report [22] in which children living in Makurdi, a north-central area of the country, were surveyed. This stunting level in this study is, however, higher than those reported by literature [25, 28] from the southwestern part of the country. The rural study location may have contributed to this disparity. These stunting values presented in this study are consistent with national estimates of stunted children in Nigeria [17]. Prevalence of underweight among children was comparable to results reported in the literature [21, 29]. The wasting levels of children in this study can be considered to be of public health significance which suggests an occurrence of severe acute illness. There was no significant difference in stunting, underweight, and wasting between boys and girls observed in this study. Several factors can be implicated as contributing to child undernutrition [30]. The Nigerian Demographic Health Survey has described these factors to include child sex, age, location, and socioeconomic status of the household which consists of factors such as parents' occupations and education with particular reference to maternal education [16]. Several other factors contributing to the high prevalence of undernutrition among children could include severe food shortage or suboptimal complementary feeding practices, and inadequate household sanitary facilities which can lead to illnesses and growth faltering [28]. Among Ethiopian children, Tessema et al. [31] stated that the prevalence of undernutrition was different across age groups of the children, which implies that each stage of growth has its peculiarity. The mean height of the women falls within the short maternal height range described by the WHO collaborative study [27, 32]. It is vital since it can be used to identify women with obstetric risks and predict reproductive outcomes [33]. Even though the maternal undernutrition in this study is low, undernourishment in women is always a situation of public health concern [34]. The more prevalent form of malnutrition among women in this study was overweight, which can be classified as a problematic situation of public health significance.

In comparison to national estimates, the values in this study confirm a rise in the prevalence of overweight/obese women of child-bearing age [17] which could indicate suggestions of nutrition transition [7, 35] which could have both short- and long-term implication for maternal and child health [10, 11, 36, 37]. If the whole population is thus considered, there is a high prevalence of undernourished children and overweight mothers which indicates the existence of a double burden of malnutrition in the same population. This observation is similar to findings locally [18] and globally [7, 14].

Contrary to usual expectations that in low-middle income settings, malnutrition is unidirectional (undernourishment), this study thus asserts that it can be in two directions. This occurrence even raises the questions of the possibility of hidden hunger being present in the same population. At the household level, this occurrence is not apparent and expectedly was not statistically significant as presented in the results. However, the existence at population level confirms reports by Popkin et al. [7, 38] who explained that variations still exist in the severity across the nations of the world. However, the existence is real, and interventions to reduce the burden should be expedited.

## 5. Conclusion and Recommendation

Prevalence of malnutrition expressed as stunting, underweight, and wasting is high among the children involved in this study, so is maternal overnutrition which is prevalent among mothers. There was no significant difference in stunting, underweight, and wasting between boys and girls. This prevalence indicates that in the same population, the presence of different forms of malnutrition exists. However, this trend is particularly worthy of note since Nigeria is still grappling with the challenges of undernutrition, and most of the interventions are usually focused in this direction. The evidence presented gives support to the growing list of reports that the existence of binary forms of malnutrition is manifesting at the household level, especially in areas of low economic status. Also, it provides a basis for strengthening any existing intervention to eradicate factors associated with double malnutrition, specifically in the South-South region and generally Nigeria. Besides, there was no relationship between the mother's BMI and any of the children's anthropometric indices.

This report, therefore, recommends that more effort be placed on active nutrition surveillance to ascertain the prevalence and periodically reassess priority challenges. We also recommend that nutrition education in the areas of healthy eating for weight control for women and interventions on infant and young child care be implemented and strengthened. These interventions should be carried out synergetically and not in parallel forms.

## Figures and Tables

**Table 1:** Age and anthropometric information of children of 6–59 months.

	Total		Boys		Girls		*p* value
	No	Mean ± SD	No	Mean ± SD	No	Mean ± SD	
Age (months)	547	30.84 ± 13.02	273	31.61 ± 13.38	274	30.08 ± 12.62	0.170
Weight (kg)		12.70 ± 3.78		13.03 ± 3.76		12.36 ± 3.76	0.041^∗^
Height (cm)	543	87.28 ± 13.39	273	88.29 ± 14.57	270	86.28 ± 12.04	0.079

^∗^Significance at *p* < 0.05.

**Table 2:** Nutritional anthropometry: height-for-age, weight-for-age, and weight-for-height for children.

Indices	Sex	−2 to +2SD (N %)	≤−2 to −3SD (N %)	<−3SD (N %)	Total (N %)
Height-for-age (stunting)	Boys	183 (30.7)	61 (10.2)	51 (8.6)	295 (49.5)
	Girls	190 (31.9)	57 (9.6)	54 (9.1)	301 (50.5)
	Total	373 (62.9)	118 (19.8)	105 (17.6)	596 (100)

Weight-for-age (underweight)	Boys	241 (40.6)	33 (5.6)	20 (3.4)	294 (49.6)
	Girls	244 (41.1)	34 (5.7)	21 (3.5)	299 (50.4)
	Total	485 (81.8)	67 (11.3)	41 (6.9)	593 (100)

Weight-for-height (wasting)	Boys	234 (42.5)	18 (3.3)	16 (2.9)	268 (48.6)
	Girls	245 (44.5)	16 (2.9)	22 (4.0)	283 (51.4)
	Total	479 (86.9)	34 (6.2)	38 (6.9)	551 (100)

>−2SD to +2SD = normal; ≤−2 to −3SD = mild/moderate; <−3SD = severe.

**Table 3:** Age and anthropometric information of mothers.

Variable	*N*	Mean ± SD	Minimum	Maximum
Age (years)	644	27.28 ± 6.85	14	50
Weight (kg)	638	58.43 ± 12.05	33	113.30
Height (cm)	641	157.52 ± 6.56	140	191.00
Body mass index (kgm^2^)	635	23.54 ± 4.60	14.55	45.45

**Table 4:** Anthropometric categories of mothers with their children.

Indices		Normal	Thin/underweight	Overweight	Obese	Total
Stunting (*p*=0.410)	Normal	247 (39.84)	36 (5.81)	100 (16.13)	38 (6.13)	421 (67.9)
	Mild/moderate	69 (11.13)	7 (1.13)	19 (3.06)	10 (1.61)	105 (16.94)
	Severe	48 (7.74)	12 (1.94)	26 (4.19)	8 (1.29)	94 (15.16)
	Total	364 (58.71)	55 (8.87)	145 (23.39)	56 (9.03)	620 (100)

Underweight (*p*=0.613)	Normal	309 (50.08)	42 (6.81)	125 (20.26)	45 (7.29)	521 (84.44)
	Mild/moderate	30 (4.86)	7 (1.13)	9 (1.46)	6 (0.97)	52 (8.43)
	Severe	23 (3.73)	6 (0.97)	11 (1.78)	4 (0.65)	44 (7.13)
	Total	362 (58.67)	55 (8.91)	145 (23.5)	55 (8.91)	617 (100)

Wasting (*p*=0.829)	Normal	321 (52.37)	47 (7.67)	130 (21.21)	48 (7.83)	546 (89.07)
	Mild/moderate	16 (2.61)	2 (0.33)	9 (1.47)	3 (0.49)	30 (4.89)
	Severe	22 (3.59)	5 (0.82)	6 (0.98)	4 (0.65)	37 (6.04)
	Total	359 (58.56)	54 (8.81)	145 (23.65)	55 (8.97)	613 (100)

## Data Availability

The data used to support the findings of this study are available from the corresponding author upon request.
